# Evaluation of 25-hydroxyvitamin D (25(OH)D) levels before and during the COVID-19 pandemic: A cross-sectional study and trend analysis involving 86,772 samples

**DOI:** 10.1371/journal.pone.0284647

**Published:** 2023-05-17

**Authors:** Mehmet Emin Arayıcı, Sila Ovgu Korkut-Uysal, Asim Leblebici, Zeynep Akcali, Deniz Tuna Edizer, Seher Kabul, Dilek Cimrin, Ender Berat Ellidokuz

**Affiliations:** 1 Department of Preventive Oncology, Institute of Health Sciences, Dokuz Eylul University, Izmir, Turkey; 2 Faculty of Engineering and Architecture, Department of Engineering Sciences, Izmir Katip Celebi University, Izmir, Turkey; 3 Department of Translational Oncology, Institute of Health Sciences, Dokuz Eylul University, Izmir, Turkey; 4 Faculty of Medicine, Department of Biochemistry, Dokuz Eylul University, Izmir, Turkey; 5 Department of Medical Services and Techniques, Medical Laboratory Techniques Program, Vocational School of Health Services, Dokuz Eylul University, Izmir, Turkey; 6 Faculty of Medicine, Department of Gastroenterology, Division of Internal Medicine, Dokuz Eylul University, Izmir, Turkey; Medical University of Gdańsk, POLAND

## Abstract

**Background:**

The aim of this paper was to evaluate the change in 25-hidroxyvitamin D (25(OH)D) levels before and during the COVID-19 pandemic.

**Methods:**

In this retrospective, cross-sectional and methodological study included 86,772 patients (18–75 years) samples who were admitted to the Izmir Dokuz Eylul University Hospital (latitude and longitude (Turkey): 27 E 09; 38 N 25, respectively) for various reasons and whose 25(OH)D levels were measured in the biochemistry unit between 2019–2020 and 2020–2021 (before and during the COVID-19 outbreak). A time series analysis of monthly averages for 25(OH)D was performed. For the purpose of seasonal study, the mean levels of 25(OH)D are grouped by years. Data were modeled in terms of 25(OH)D levels using the MATLAB Curve Fitting Toolbox.

**Results:**

There was no significant difference between the sexes according to 25(OH)D levels (p>0.05). 25(OH)D levels were significantly higher in the summer months and lower in the winter months (p<0.001). When comparing the spring months, 25(OH)D levels in 2020 (18 ± 10) were found to be significantly lower than in 2019 (22 ± 12) (p<0.001); on the contrary, when examined based on the summer, autumn, and winter months, it was determined that 25(OH)D levels increased in 2020 (summer: 25 ± 13, autumn: 25 ± 14, and winter: 19 ± 10) compared to 2019 (summer: 23 ± 11, autumn: 22 ± 10, and winter: 19 ± 11) (p<0.001). In the estimates curve obtained with an error margin of 11% in the time series analysis, it was estimated that the 25(OH)D averages after the pandemic would be similar to those before the pandemic.

**Conclusions:**

Restrictions, partial or complete closures, and curfews can significantly affect individuals’ 25(OH)D levels during the COVID-19 outbreak. There is a need for multicenter studies with larger populations covering different regions to strengthen and support our results.

## Introduction

SARS-CoV-2 (severe acute respiratory syndrome coronavirus 2), which caused the COVID-19 pandemic, exerts its effect by infecting pulmonary epithelial cells through the ACE-2 (angiotensin-converting enzyme-2) receptor [1]. While SARS-CoV-2 causes damage to the pulmonary epithelium, it also infects macrophages using ACE-2 receptors and leads to their activation [2]. Macrophages, neutrophils, and T cells can be activated through sustained elevation of important cytokines such as interleukin (IL)-1, IL-6, and tumor necrosis factor (TNF) alpha. Eventually, type 2 pneumocyte apoptosis may be induced and patients may directly face acute respiratory distress syndrome (ARDS) [2]. At this point, host responses can be enhanced in some cases by the overwhelming expression of proinflammatory cytokines. This "cytokine storm" can be blamed for some of the serious events of COVID-19, such as ARDS [3,4].

Vitamin D is defined as a steroid hormone produced in the body by exposure of the human skin to UV (ultraviolet) B rays and has important roles in calcium and phosphorus metabolism as well as bone mineralization [5,6]. Subcutaneous production can be described as the major source of vitamin D synthesis, although foods such as dairy products, fish oil, and liver also contain noticeable amounts of vitamin D [6–8]. Synthesis and availability of vitamin D depend on many important factors, including exposure to sunlight, latitude, the season of the year, the hour of the day, pigmentation of the skin, age, sex, and body mass index [7]. Vitamin D binds to nuclear vitamin D receptors called VDRs during cellular events, forming a VDRE. In this case, the activated VDR can attach to the cathelicidin gene promoter VDRE and thus cause the host to initiate defense against some viral infections [9]. Vitamin D can also directly affect the innate immune system through the expression of lysosomal enzymes and the release of nitric oxide [5,9]. In both cases, Vitamin D directly contributes to the fight against infection. Thus, vitamin D plays an immunomodulatory role in SARS-CoV-2 infection by suppressing the adaptive immune system of respiratory epithelial cells [5–9].

It is a well-known fact that Vitamin D deficiency is a risk factor for many diseases, such as autoimmune diseases [10], cardiovascular diseases [11], and cancer [12,13]. Vitamin D deficiency in individuals has been clearly demonstrated in several studies conducted in many countries, including Turkey [14–17].

In recent years, there has been an increasing interest in studies on vitamin D [18–27]. Several prior studies have focused on both disease pathogenesis and the etiology of vitamin D [18–23]. Some studies focused on seasonal changes in vitamin D levels and the relationship between vitamin D and diet [24–27]. Degerud et al. (2016) investigated the seasonal 25-hydroxyvitamin D (25(OH)D) levels in cardiovascular diseases [28]. In another study conducted by Zgaga et al. (2022), statistical power was evaluated in randomized controlled trials investigating 25(OH)D [29]. Additionally, a 2015 Irish study evaluated at the trend of 25(OH)D over 20 years from 1993 to 2013 [30]. Similarly, in another study, trends in 25(OH)D results in a total of 40,307 samples were modeled using a time series analysis [31]. The purpose of this paper was to evaluate the change in 25(OH)D levels before and during the COVID-19 pandemic. In addition, the seasonal variation in 25(OH)D levels during the COVID-19 period was examined not only biologically but also mathematically, and the seasonal variation in 25(OH)D levels was modeled mathematically.

## Materials and methods

In this retrospective, the cross-sectional and methodological study included 125,643 patients (18–75 years) who were admitted to the Izmir Dokuz Eylul University Hospital (latitude and longitude (Turkey): 27 E 09; 38 N 25, respectively) for various reasons and whose 25(OH)D levels were measured in the Biochemistry unit between 2019–2020 and 2020–2021 (before and during the COVID-19 outbreak). It has been accepted that 2020 reflects the period of the COVID-19 pandemic, while 2019 reflects the non-pandemic period. The dependent variable of the study was 25(OH)D level, and the independent variables were defined as age, sex, seasons, and months. The months in the seasons are defined as follows: the spring months are March-April-May; the summer months are June-July-August; the autumn months are September-October-November; and the winter months are December-January-February. This study was approved by the Dokuz Eylul University Non-Interventional Research Ethics Committee (Date: 05.01.2022, Approval No: 2022/01-26). Written informed consent was obtained from the patients, and all techniques followed the Declaration of Helsinki.

### Vitamin D total analytical properties

The vitamin D total test was measured in vitro for the quantitative determination of a total of 25(OH)D in human serum and plasma (EDTA, lithium-heparin, sodium-heparin) using ADVIA Centaur XP systems (Siemens Healthcare Diagnostics, Tarrytown, NY, USA) [32]. The ADVIA Centaur vitamin D test is measured with the chemiluminescence immune method. It is a competitive immunoassay using an anti-25(OH)D monoclonal mouse antibody labeled with acridinium ester (AE) and a fluorescently labeled vitamin D analog. Analysis can be monitored with the REF 10493589 ADVIA Centaur vitamin D Calibrator. Three levels of REF 10632229 ADVIA Centaur Vitamin D Control are used as internal quality control. The range of 25(OH)D values is as follows: <20 ng/mL is considered deficient; 21–29 ng/mL is considered insufficient; and 30–100 ng/mL is considered sufficient [33].

### Performance characteristics of the analysis

Measuring range: The ADVIA Centaur Vitamin D test (Siemens Healthcare Diagnostics, Tarrytown, NY, USA) [32] measures 25(OH)D in the concentration range from 4.2 to 150 ng/ml (10.5 to 375 nmol/l); Sensitivity: The functional sensitivity of the ADVIA Centaur Vitamin D test is 3.3 ng/ml (8.3 nmol/l); Repeatability (Precision): 4.9% Within-Run 5.4% Between-Runs; Method Comparison: ADVIA Centaur Vitamin D = 1.15 (LC/MS/MS) + 0.7 ng/ml, r = 0.91. In the verification study performed in the laboratory, the total error was found to be 13%.

### Description of the dataset

As mentioned earlier, the dataset was taken by "The Biochemical Laboratory of Dokuz Eylul University". The observed raw data includes 125,643 analysis results. Moreover, the features of the dataset contain information about age, the date of birth, sex, the date of the analysis, and the 25(OH)D level of the patients. Among these features, "sex" is a categorical value, the date of analysis and the date of birth are time series, and the rest of them are numerical values.

The raw data has been cleaned elaborately with the consensus of the authors. First, the unnecessary columns are removed. This step is followed by limiting the study to the range of 18–75 years due to a potential bias that may occur from age. Then, both the incidental values and the null values have been removed. Additionally, the outliers are also removed. To investigate the seasonal effects of COVID-19 on the levels of 25(OH)D, the ’Date of Analysis’ time series has been manipulated to convert it into "Year" and "Month". Finally, the years are grouped by considering the mean of the 25(OH)D Score.

After the cleaning process has been completed successfully, the dataset contains 86,772 samples and five features such as "age", "sex", "25(OH)D score", "year" and "month".

### Mathematical model

In this study, the collected dataset contains both numerical and categorical values. Therefore, the first aim has been designed to study the categorical values priorly. For this purpose, the significance levels of the mean score of 25(OH)D were carried out for both sexes in a month. Moreover, for a continuous mathematical model, the age column was also excluded. Thereafter, for the purpose of seasonal study, the mean levels of 25(OH)D are grouped by years.

Vitamin-D levels have a wave-curved form. It is worth noting that the results can vary from data to data. The following functions can be utilized to describe a model for such a curve:

$$Proposed\;Model\;1\;(PM1):\;{\hat{L}}_{Vit-D}(t)=\sum_{i=1}^{N_{e}}a_{i}\;\sin\left(b_{i}t+c_{i}\right)$$ (1)

$$Proposed\;Model\;2\;(PM2):\;{{\hat{L}}_{Vit-D}(t)=a}_{0}+\sum_{j=1}^{N_{e}}a_{j}cos(j\omega t)+b_{j}\;\sin\left(j\omega t\right)$$ (2)

where *a*_*i*_, *b*_*i*_, *c*_*i*_ and *ω* are constants. Moreover, ${\hat{L}}_{Vit-D}(t)$ represents the approximated value of 25(OH)D levels where *t* stands for time (in months). Furthermore, *N*_*e*_ denotes the number of terms of the approximation.

The rearranged data were randomly divided into training (70%) and test (30%) sets. The goodness of the models was controlled by *R*^2^ and Adjusted-*R*^2^ (A_*R*^2^) such that:

$$R^{2}=1-\frac{SSE}{SST}$$ (3)

$$A\_R^{2}=1-\left(\frac{n-1}{n-p}\right)\frac{SSE}{SST}$$ (4)

Notice that SSE and SST denote the sum of squared error and the sum of the squared total, respectively. Additionally, n is the number of observations, and p is the number of regression coefficients.

### Statistical analysis

In the evaluation of the data, descriptive statistics, means, median values (interquartile range (IQR)), and standard deviations (sd) of the patients were calculated. Compliance with the normal distribution was checked with the Kolmogorov-Smirnov test. Student’s t-test and ANOVA were used to examine the differences between continuous variables with normal distribution. In the evaluation of continuous data not suitable for normal distribution, Mann Whitney U test and Kruskal Wallis test were used according to their suitability. All computational studies were performed using MATLAB software (version 2021b). For describing the model, the MATLAB Curve Fitting Toolbox has been used. In so doing, SSE has been optimized by the Trust-Region algorithm, where all the other parameters are selected as default. The SPSS (version 24.0) and R (version 4.1.3) package programs were also used for the analysis and visualization of statistical data. The statistical significance level was determined as a two-tailed p<0.05.

## Results

A total of 86,772 samples were included in this study, and 58,545 (67.5%) of the study group were women. The median age of the research group was 54 (IQR, 40–65) and the mean age was determined to be 52 ± 16 years. The median and mean 25(OH)D levels at the time of measurement were also calculated at 20 (IQR, 14–27) ng/ml and 22 ±12 ng/ml, respectively. When the research group was grouped in terms of 25(OH)D levels, it was determined that 44,103 (50.8%) participants were deficient, 27,689 (31.9%) were insufficient, and only 14,974 (17.3%) were sufficient. The descriptive statistics of the research group are summarized in Table 1.

**Table 1:** Descriptive features of the research group.

Variables	Total (n = 86,772)
Sex, n (%) Women Men	58,545 (67.5) 28,227 (32.5)
Age, median (IQR), year	54 (40–65)
Age group, n (%) ≤18–30≤ <30–45≤ <45–60≤ <60–75≤	11,118 (12.9) 17,381 (20) 27,127 (31.3) 31,070 (35.8)
25(OH)D level, mean (SD), ng/ml	21.6 ±11.5
Year groups, n (%) 2019 2020 2021	43,238 (49.9) 17,716 (20.4) 25,717 (29.7)
25(OH)D group, n (%), ng/ml <20 21–29 >30	44,103 (50.8) 27,689 (31.9) 14.974 (17.3)

IQR: interquartile range, SD: standart deviation, 25(OH)D: 25-Hidroxyvitamin D.

**Fig 1:**
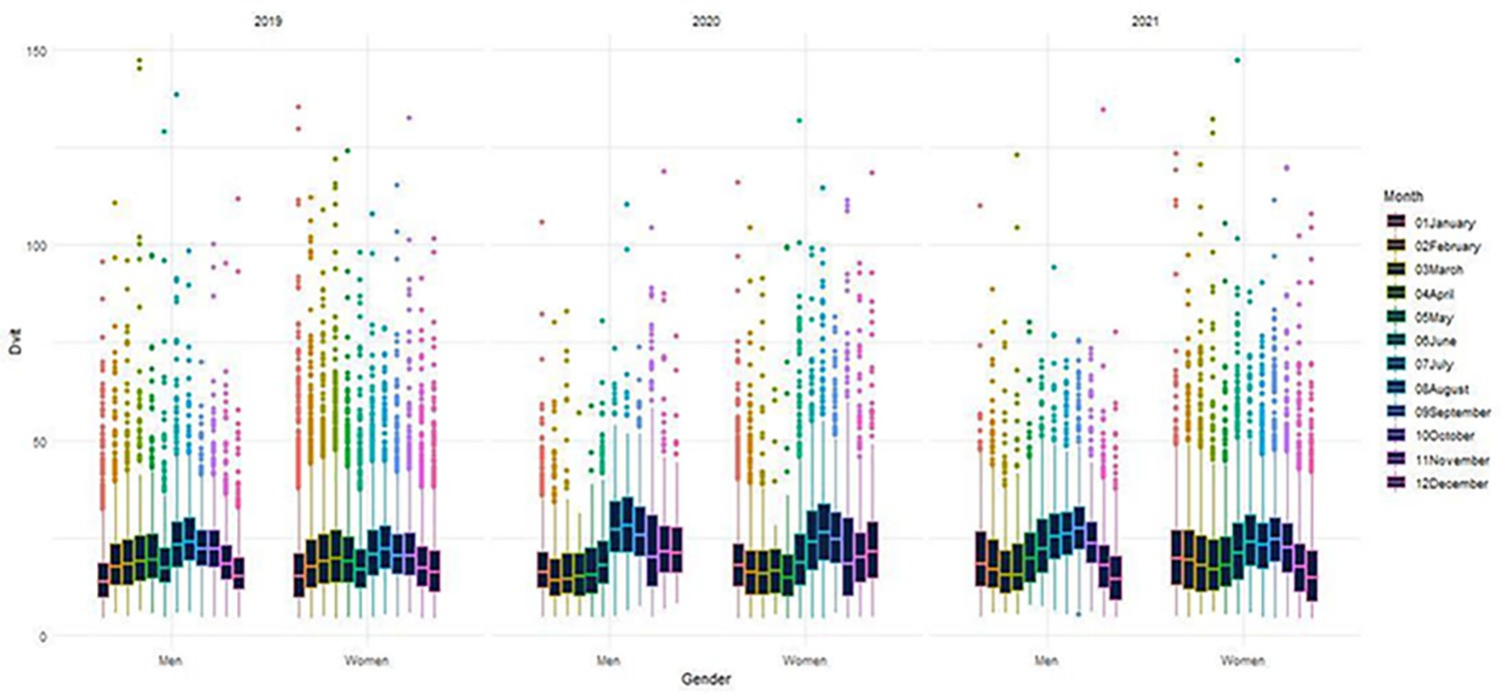
Distribution of 25(OH)D levels by sex and month.

**Fig 2:**
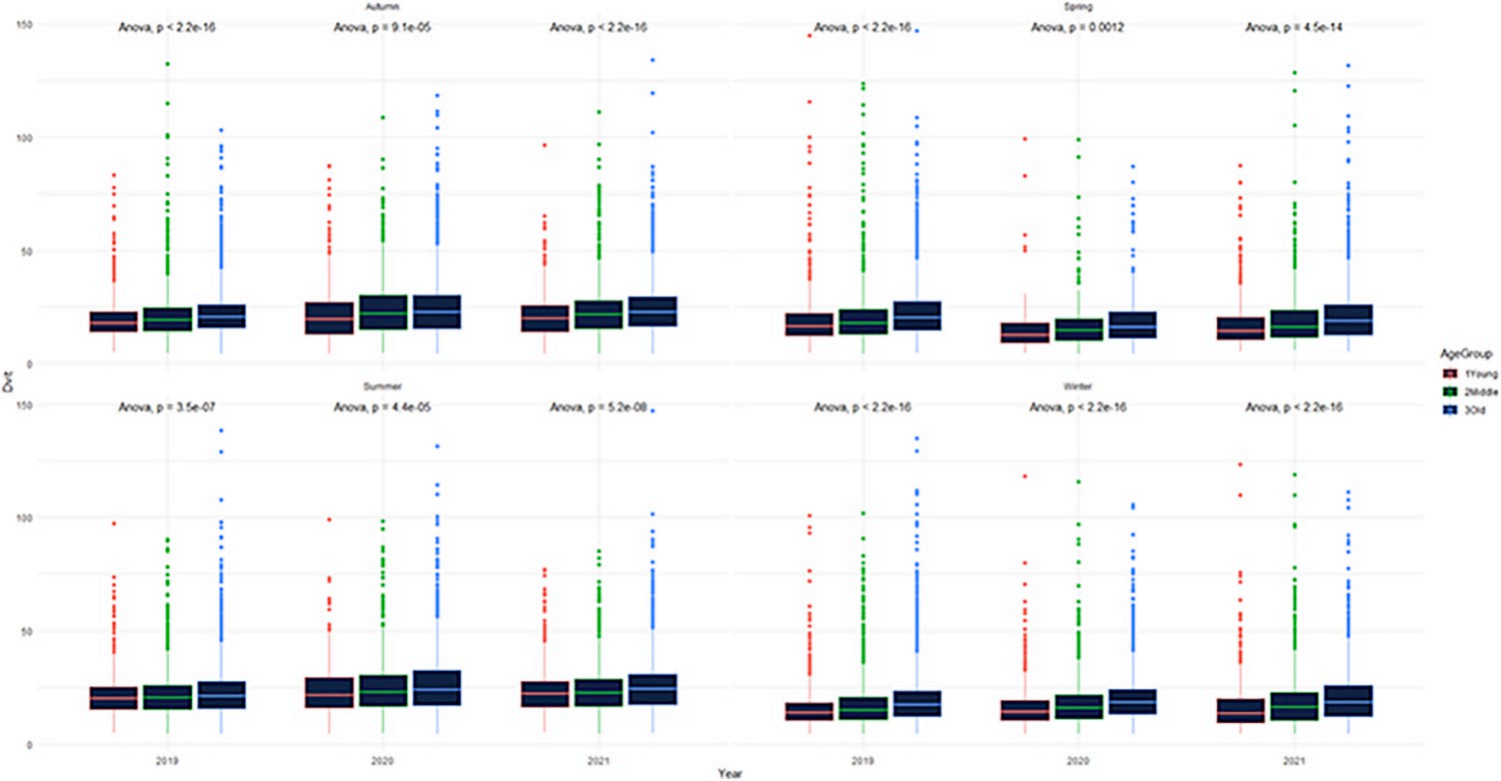
Distribution of 25(OH)D levels by season and age groups.

**Fig 3:**
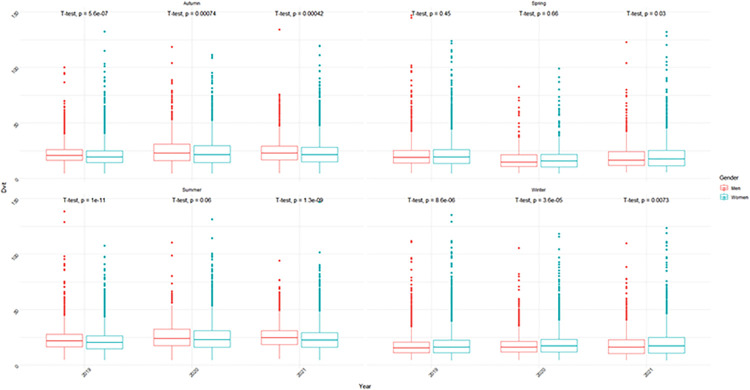
Distribution of 25(OH)D levels by season and sex.

**Fig 4:**
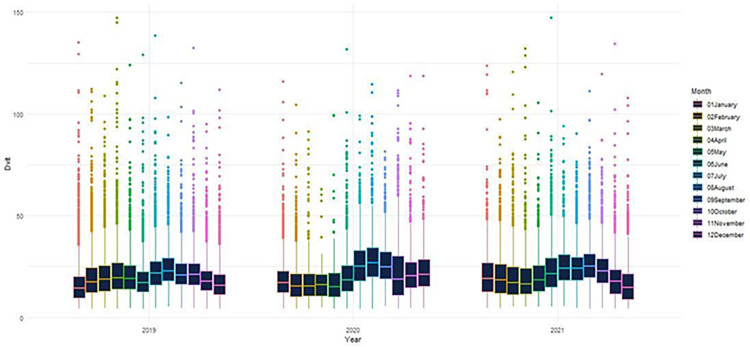
Distribution of 25(OH)D levels by years and months.

The relationship between 25(OH)D levels and sex was investigated. There is no significant difference between the sexes according to 25(OH)D levels (p>0.05) (Fig 1). The association between vitamin D levels and age group was also investigated. According to the analysis results, it has been found that 25(OH)D levels increase significantly as age increases (p<0.001) (Fig 2). When 25(OH)D levels were examined according to the seasons, as shown in Figs 2 and 3, it was determined that 25(OH)D levels were significantly higher in the summer months and lower in the winter months (p<0.001). 25(OH)D levels were found to be higher in autumn than in spring, which can be described as the transition season (p<0.001) (Fig 3). In addition to these, seasonal 25(OH)D changes according to years were also evaluated. According to the test results, when comparing the spring months, 25(OH)D levels in 2020 (18 ± 10) were found to be significantly lower than in 2019 (22 ± 12) (p<0.001); on the contrary, when examined based on the summer, autumn, and winter months, it was determined that 25(OH)D levels increased in 2020 (summer: 25 ± 13, autumn: 25 ± 14, and winter: 19 ± 10) compared to 2019 (summer: 23 ± 11, autumn: 22 ± 10, and winter: 19 ± 11) (p<0.001) (Fig 4).

**Fig 5:**
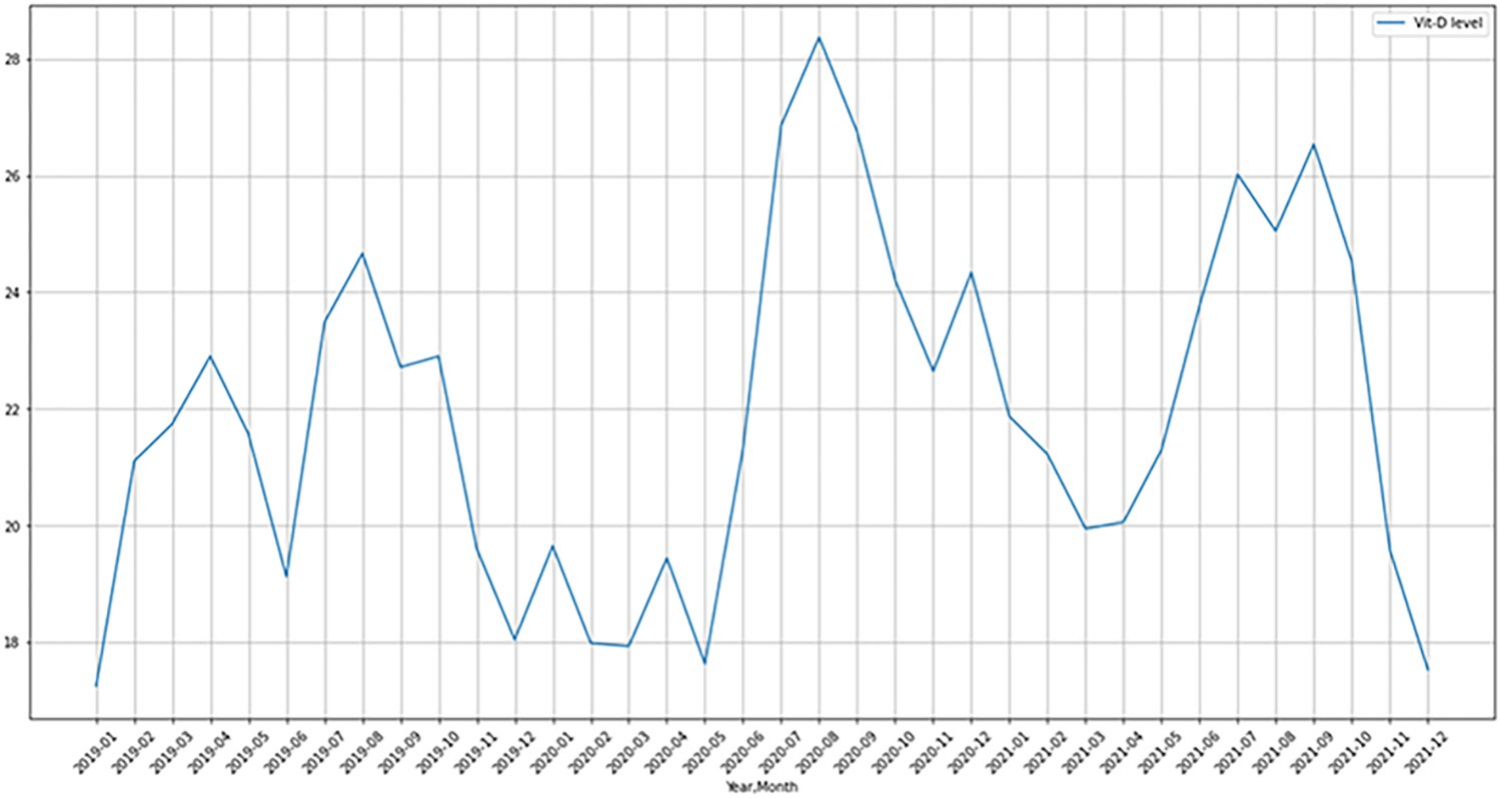
The trend line of the mean 25(OH)D levels in years.

From a mathematical point of view, Fig 1 emphasizes that the mean 25(OH)D levels in both men and women have a similar trend line. That is, the sex column can be considered negligible. Fig 5 is evidence that the 25(OH)D levels have a wave-curved form. Once all the preprocessing has been fulfilled, the trend line of the 25(OH)D levels in years is presented in Fig 5. In this study, two mathematical models have been suggested to approximate the cleaned dataset (70%). The results are listed in Table 2.

**Table 2:** Comparisons of PM1 and PM2 in terms of N_e_.

	PM1 (sine)				PM2 (fourier)			
	*N*_*e*_ = 3	*N*_*e*_ = 4	*N*_*e*_ = 5	*N*_*e*_ = 6	*N*_*e*_ = 3	*N*_*e*_ = 4	*N*_*e*_ = 5	*N*_*e*_ = 6
***R*** ^**2**^	0.82	0.85	0.92	0.96	0.80	0.84	0.94	0.91
**A**_***R***^**2**^	0.73	0.72	0.81	0.89	0.73	0.75	0.89	0.81
**#** of coefficients	9	12	15	18	8	10	12	14

As seen in Table 2, as *N*_*e*_ increases the accuracies of the models are getting better. However, the goodness of the PM2 interrupted its consistency when *N*_*e*_ = 6. This probably happened because of the over-estimation. Considering the accuracies and the number of coefficients altogether, the PM2 for *N*_*e*_ = 5 seems much preferable than those PM1. Thus, the mathematical model of the estimations of levels of 25(OH)D is

$${{\hat{L}}_{Vit-D}(t)=a}_{0}+\sum_{j=1}^{N_{e}}a_{j}\;cos(j\omega t)+b_{j}\;\sin\left(j\omega t\right)$$ (5)

$$\begin{array}{llll} \;a_{0}=21.99 & a_{1}=2.203 & a_{2}=-1.039 & a_{3}=-0.1941\\ \;a_{4}=0.13 & a_{5}=1.003 & b_{1}=0.3411 & b_{2}=-3.192\\ b_{3}=-0.06879 & b_{4}=1.575 & b_{4}=0.3129 & \omega =0.2592 \end{array}$$ (6)

S1 Fig indicates how fits the proposed model to the raw data set.

After the estimated values are computed the proposed model has been tested on the test set. For a reliable test, the root means square error (RMSE) and the relative error (RE) have been considered.

$$RMSE={\left(\frac{1}{11}\sum_{i=1}^{11}{\left|L_{Vit-D}(t_{i})-{\hat{L}}_{Vit-D}(t_{i})\right|}^{2}\right)}^{1/2}$$ (7)

$$RE=\frac{\sum_{i=1}^{11}\left|L_{Vit-D}(t_{i})-{\hat{L}}_{Vit-D}(t_{i})\right|}{\sum_{i=1}^{11}\left|L_{Vit-D}(t_{i})\right|}$$ (8)

where *L*_*Vit*−*D*_(*t*_*i*_) is the value of raw data at *t* - *t*_*i*_, and ${\hat{L}}_{Vit-D}(t_{i})$ is the predicted data values, *i* = 1,2,..11.

For the proposed model the errors are as follows: RE = 0.1110 and RMSE = 2.8839. This guarantees that the proposed model is a good agreement with the test data with an 11% error margin. A further investigation has been done for predicting the 25(OH)D levels in the upcoming months. Before ending the section, the graph of the future predictions has been illustrated in S2 Fig. In the estimates curve obtained with an error margin of 11% in the time series analysis, it was estimated that the 25(OH)D averages after the pandemic would be similar to those before the pandemic.

## Discussion

Previous studies have revealed that approximately more than half of individuals measured 25(OH)D have vitamin D deficiency (<20 ng/mL) [15,17,34]. These rates vary according to geographical region, latitude and characteristics of the patient population [14,15]. In a meta-analysis conducted by Alpdemir et al., which included 40 studies examining the deficiency in Vitamin D levels in Turkey, it was reported that the prevalence of vitamin D deficiency was 63.0% (95% CI: 58.9–66.6) for the general population [14]. In this meta-analysis, it was underlined that the risk of vitamin deficiency is higher especially in neonates, pregnant women and adult women [14]. Similar to other studies, 25(OH)D levels were found to be deficient (20 ng/mL) in 50.8% of individuals, insufficient (20–29 ng/mL) in 31.9% of individuals, and sufficient (>30 ng/mL) in 17.3% of individuals in this research.

The results of previous studies revealed that there was no clear difference between the genders in terms of vitamin D deficiency. Some studies demonstrated that vitamin D deficiency was more common in females, whereas others stated that it was more common in males [35,36]. In a study conducted in the Aegean Region of Turkey by Hekimsoy et al., it was reported that 25(OH)D deficiency was more common among females (78.7%) than males (66.4%, p < 0.05) [15]. Our data show that there was no statistically significant difference between males and females.

Several prior reports indicate that older age is a risk factor for vitamin D deficiency [34,35,37]. Even healthy elderly people are advised to take higher doses of vitamin D [38]. Our analysis, on the contrary, points out that vitamin D levels increase in older age groups. This may be related to higher vitamin D supplementation in older age groups in this pandemic period.

When the spring months were examined, we discovered that 25(OH)D levels were significantly lower in 2020 than in 2019 (p<0.001). The rapid increase in COVID-19 cases and the emergence of restriction measures can be shown as the reasons for the decrease in 25(OH)D levels. Because the COVID-19 outbreak was declared a pandemic by the World Health Organization (WHO) on March 11, 2020 [39,40]. On the same date (11 March 2020), the first case of COVID-19 was detected in Turkey [41]. On April 3, entrances and exits to the city were banned in 30 metropolitan cities in Turkey for 15 days, and later, the capacity of restrictions was expanded across the country day after day [42]. Therefore, due to restrictions, people were not exposed to getting enough sunlight, so 25(OH)D levels may have dropped.

We compared the years 2020 and 2021 and discovered that 25(OH)D levels increased significantly in 2021 compared to 2020 (p<0.001). Similarly, a study conducted in Ireland evaluated laboratory-based samples of circulating 25(OH)D before and during the COVID-19 pandemic [43]. In this study, it was reported that the level of 25(OH)D increased by 2.8 nmol/L (61.4, 95% CI 61.5 to 61.7 vs 58.6, 95% CI 58.4 to 58.9, p<0.001) during the COVID-19 pandemic [44]. In various randomized controlled and observational studies, it has been emphasized that the use of various types and amounts of 25(OH)D supplements may have a protective effect against COVID-19 [45–49]. Therefore, this rise in 25(OH)D levels suggests that it may be due to the increase in the use of Vitamin D supplements, as they may be protective against COVID-19 or reduce its side effects.

When 2019 and 2021 are compared, we found that although 25(OH)D levels in 2021 were higher than in 2020, they were lower than in 2019. There was a second pandemic wave during the COVID-19 period [44]. This decrease in 25(OH)D may have been caused by restrictions in the second pandemic wave of COVID-19. However, this decrease in 25(OH)D levels is relatively less than the decrease in the onset of COVID-19.

Mathematical models are used as a tool not only to understand the truth behind real-life problems but also to develop measures to be taken to solve these problems. In this study, even though two mathematical models have been suggested, a more convenient one has been selected to analyze the dataset. Therefore, considering all the presented tables and figures together, the described mathematical model fits well with the real data.

There were some limitations to this research. The first is that all the included data was obtained from the hospital database. While it is an advantage to have data from a large hospital database in the region, data from a single center may not reflect the population’s 25(OH)D levels. Likewise, hospital access has decreased because of restrictions. Due to restrictions on access to health services in our country, as in many countries, hospital admissions were limited to emergency patients during the COVID-19 outbreak. Therefore, the characteristics of patients from the COVID-19 pandemic period may differ significantly from patients from other periods. This may also involve a certain amount of bias in determining the actual 25(OH)D levels in the population. Similarly, another limitation is that it is not known how many of the patients were admitted to the hospital due to COVID-19 during the pandemic. Another limitation is that the study was carried out in a relatively sunny region of Turkey, and this may be misleading as to the actual 25(OH)D levels in the country. In addition to all this, from the mathematical point of view, the obtained coefficient can vary due to the random division of the training and test sets. This means that the returned values of levels of 25(OH)D can change according to the coefficient.

## Conclusions

This study was undertaken to examine the change in 25(OH)D levels before and during COVID-19 pandemic. In addition, the trend of change in 25(OH)D levels was mathematically modeled. Restrictions, partial or complete closures, and curfews can significantly affect individuals’ 25(OH)D levels during the COVID-19 outbreak. The use of masks during the pandemic period may also impact vitamin D levels by reducing the body surface area exposed to solar UVB. In the estimates curve obtained with an error margin of 11% in the time series analysis, it was estimated that the 25(OH)D averages after the pandemic would be similar to those before the pandemic. Therefore, possible decreases in 25(OH)D in individuals may cause an increase in the incidence of 25(OH)D-related diseases. 25(OH)D is an essential and necessary vitamin for individuals of all ages. As such, vitamin D is a vital component in the human body that acts as both a vitamin and a steroid hormone. Thus, policies must be created to raise awareness of the community about 25(OH)D in cases of partial or complete closure [50–52]. There is a need for multicenter studies with larger populations covering different regions to strengthen and support our results.

## Supporting information

S1 Fig Comparisons of the estimations of 25(OH)D levels of the proposed model 1 and the real 25(OH)D values. (DOCX)

S2 Fig Future estimation of levels 25(OH)D with the help of the proposed model. (DOCX)
