# Supplementary material for: Evaluation of 25-hydroxyvitamin D (25(OH)D) levels before and during the COVID-19 pandemic: A cross-sectional study and trend analysis involving 86,772 samples
Source: PLoS One. 2023 May 17;18(5):e0284647. doi: 10.1371/journal.pone.0284647 (PMC10191318; doi:10.1371/journal.pone.0284647)
Supplement: S1 Fig — (DOCX) [file pone.0284647.s001.docx]

**Supporting information 1**


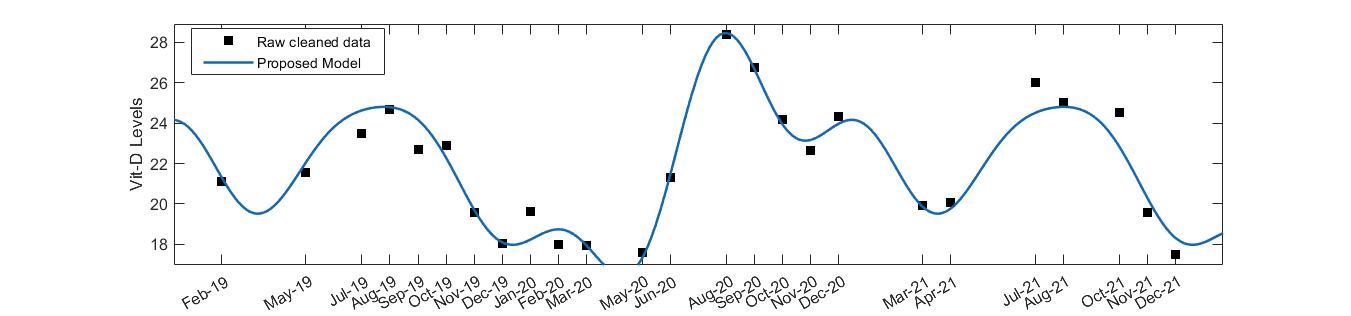


**Fig S1.** Comparisons of the estimations of 25(OH)D levels of the proposed model 1 and the real 25(OH)D values
